# Gap Opening in Double-Sided Highly Hydrogenated Free-Standing Graphene

**DOI:** 10.1021/acs.nanolett.2c00162

**Published:** 2022-03-16

**Authors:** Maria Grazia Betti, Ernesto Placidi, Chiara Izzo, Elena Blundo, Antonio Polimeni, Marco Sbroscia, José Avila, Pavel Dudin, Kailong Hu, Yoshikazu Ito, Deborah Prezzi, Miki Bonacci, Elisa Molinari, Carlo Mariani

**Affiliations:** † Physics Department, Sapienza University of Rome, Piazzale Aldo Moro 5, 00185 Rome, Italy; ‡ Synchrotron SOLEIL, Université Paris-Saclay, Saint Aubin, BP 48, 91192 Gif sur Yvette, France; ¶ School of Materials Science and Engineering and Institute of Materials Genome & Big Data, Harbin Institute of Technology, Shenzhen 518055, P.R. China; § Institute of Applied Physics, Graduate School of Pure and Applied Sciences, University of Tsukuba, Tsukuba 305-8573, Japan; ∥ S3, Istituto Nanoscienze-CNR, Via Campi 213/A, 41125 Modena, Italy; ⊥ Dipartimento di Scienze Fisiche, Informatiche e Matematiche (FIM), Università degli Studi di Modena e Reggio Emilia, 41125 Modena, Italy

**Keywords:** graphane, nanoporous graphene, hydrogen functionalization, spectromicroscopy, density functional theory, GW calculations

## Abstract

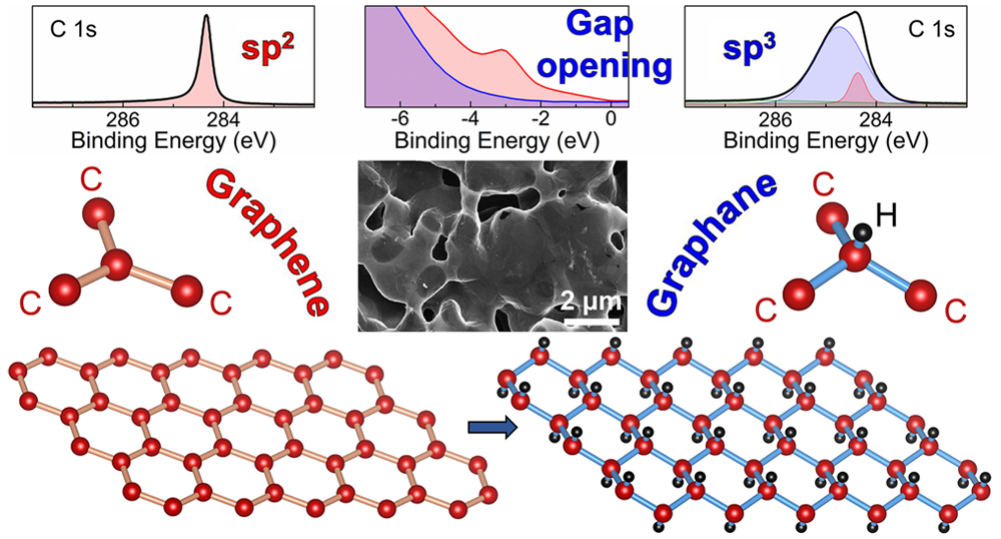

Conversion of free-standing graphene into pure graphane—where each C atom is sp^3^ bound to a hydrogen atom—has not been achieved so far, in spite of numerous experimental attempts. Here, we obtain an unprecedented level of hydrogenation (≈90% of sp^3^ bonds) by exposing fully free-standing nanoporous samples—constituted by a single to a few veils of smoothly rippled graphene—to atomic hydrogen in ultrahigh vacuum. Such a controlled hydrogenation of high-quality and high-specific-area samples converts the original conductive graphene into a wide gap semiconductor, with the valence band maximum (VBM) ∼ 3.5 eV below the Fermi level, as monitored by photoemission spectromicroscopy and confirmed by theoretical predictions. In fact, the calculated band structure unequivocally identifies the achievement of a stable, double-sided fully hydrogenated configuration, with gap opening and no trace of π states, in excellent agreement with the experimental results.

## Introduction

Maximum storage of hydrogen in carbon-based materials is ideally achieved in graphene by forming the so-called graphane, where each carbon atom in the honeycomb lattice is bound to hydrogen with alternately up and down sp^3^ distorted bonds. In graphane, the conjugation of graphene π electrons is thus disrupted, leading to an insulating behavior with band gap predicted to depend on the H chemisorption configuration.^1,2^

Experimentally, however, only a low H storage capacity has been reached so far (∼36 at. %) on single-layer graphene^3−14^ with non-negligible contamination and defects. The maximum H uptake depends on both the morphology of graphene specimens (substrate-supported, transferred flakes, etc.) and the hydrogenation methods. Several attempts to incorporate a high percentage of hydrogen have been carried out in the last decades, exploiting a variety of strategies on different samples. Hot^6,7,10^ and cold^4,14^ plasma deposition and molecular H_2_ high-temperature cracking^5,9^ were applied to exfoliated graphene layers,^3,10^ to chemical-vapor-deposition (CVD) grown flakes,^6,15,16^ or even to metal–supported samples,^5,8,9,11,17^ reaching at most a partial hydrogenation of monolayer graphene, with an upper limit of H uptake Θ ∼ 36 at. %,^14^ while an almost stoichiometric bulk graphane has been obtained from halogenated reduced wrinkled and layered graphenes.^18^ The limit of hydrogenation in single-layer graphene can be due to several concurrent drawbacks, such as oxygen contamination, the influence of the substrate, and the presence of defects/edges in graphene flakes (either pre-existing or induced by the hydrogenation itself).

A crucial challenge to fully exploit graphene for hydrogen storage is to employ defect-free graphene specimens with very high specific surface area, where hydrogen can adsorb strongly enough on the surface as to form a thermodynamically stable arrangement, achieving an ideal graphane pattern. Nanoporous graphene (NPG)—constituted by a compact, bicontinuous interconnected 3D arrangement of high-quality graphene veils, composed of one to a few weakly interacting layers^19,20^—can present great advantages to achieve a high uptake of hydrogen in graphene. The free-standing, curved structure at the submicrometer scale, with intrinsically smooth rippling, can foster hydrogen chemisorption, favored by the increased electron affinity of hydrogen and the energy barrier decrease in the convex regions^21^ induced by the pull out of the C atom toward the H proton.^22,23^

We here exploit high-quality NPG samples, with high specific area (1000 m^2^/g)^24−26^ and very low density of defects, together with in situ, highly controlled hydrogenation by H_2_ cracking in the ultrahigh vacuum (UHV) condition. In this way, we can control the absence of contamination of the sample during the H exposure and realize a thermodynamically stable prototype of semiconducting graphane. A spectromicroscopy photoemission study, combined with state-of-the-art theoretical predictions, demonstrates an unprecedentedly high H uptake in NPG, accompanied by a spectral electronic density of states as predicted for ideal graphane, achieving for the first time an almost complete saturation in fully free-standing graphene of the available C sites with hydrogen.

## Results and Discussion

**Figure 1:**
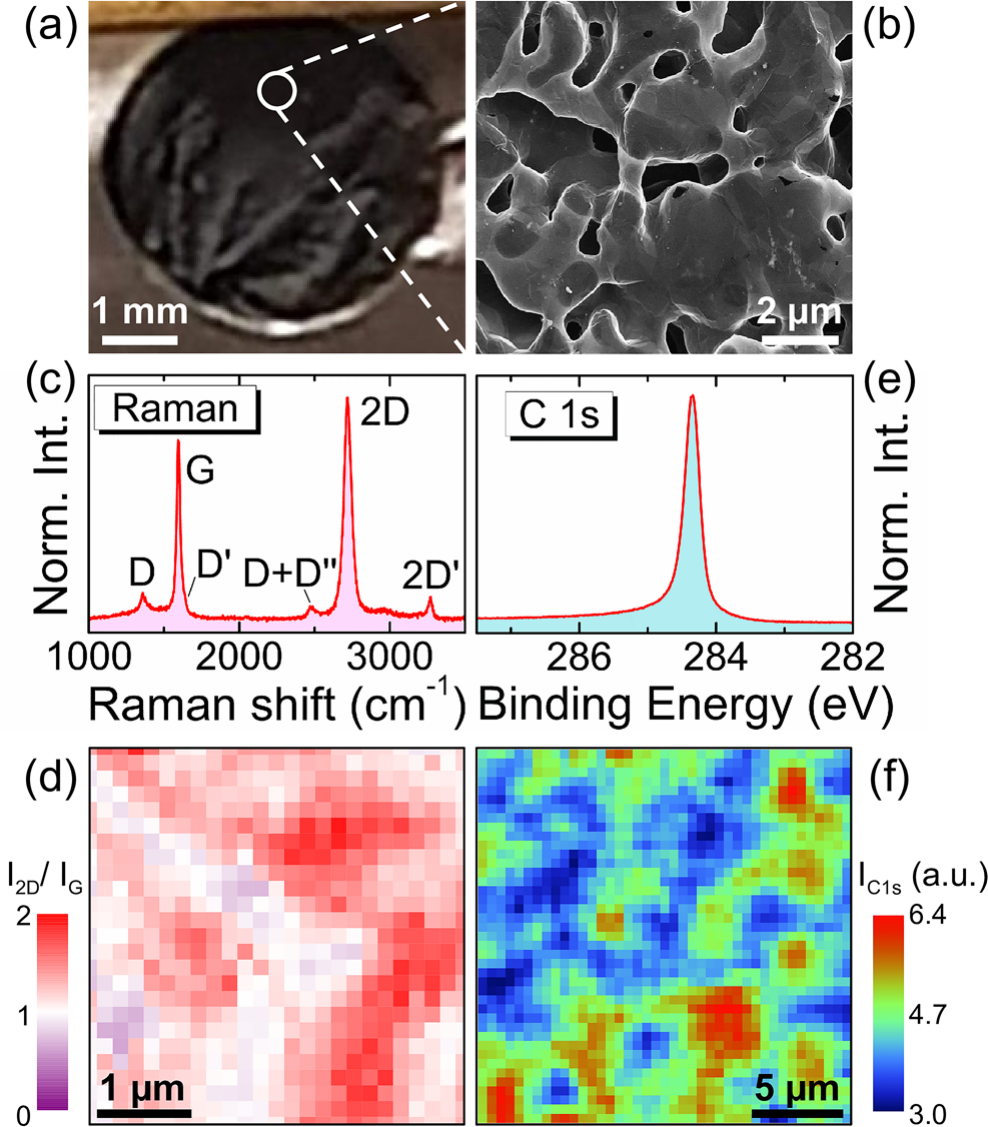
(a) Optical picture of an NPG sample. (b) SEM image of NPG (10 × 10 μm^2^). (c) Micro-Raman spectrum taken with a 100× objective (Gaussian laser spot size with σ = 280 nm). (d) Spatially resolved micro-Raman map of the 2D/G band intensity ratio, 4 × 4 μm^2^ image formed by 167 × 167 nm^2^ pixels. (e) Spatially integrated C 1s XPS spectrum, taken with 350 eV photon energy. (f) Spatially resolved micro-X-ray photoelectron spectroscopy (micro-XPS) map of the C 1s core level intensity (282–288 eV energy range), 20 × 20 μm^2^ image formed by 500 × 500 nm^2^ pixels.

Figure 1a displays an optical picture of a free-standing NPG sample (∼0.5 cm in diameter) with large surface area density (1000 m^2^/g)^24−26^ arranged in a compact 3D structure; see Experimental and Computational Methods for details on the sample preparation. Scanning electron microscopy (SEM) imaging (Figure 1b) zoomed at a mesoscopic level (10 × 10 μm^2^) unveils the porous structure (pore size in the submicrometer to few micrometer range) constituted by a folded tubular graphene sheet with continuous almost flat areas and interconnected channels, with some wrinkles and without frayed edges.^24−26^ A detailed analysis of the NPG microscopic structure at the atomic scale, reported elsewhere,^19^ revealed moiré superstructures due to suspended graphene layers with misoriented (turbostratic) stacking in some regions. Within this macroscopic 3D graphene architecture, the microscopic structure preserves all the 2D graphene hallmarks.^19^

A representative micro-Raman spectrum (see Experimental and Computational Methods) of the NPG sample is shown in Figure 1c. The measurements were performed with moderate power densities (∼20 kW/cm^2^), analogous to those employed for other suspended 2D systems such as hexagonal boron nitride bubbles,^27^ and after verifying that no detrimental effects were induced by the laser beam on the NPG sample. The Raman spectrum presents a low intensity of the defect-activated “D” Raman peak if compared to the “*G*” peak, associated with the doubly degenerate (iTO and LO) phonon modes at the Brillouin zone center. More importantly, the Raman spectrum shows a high intensity of the “2D” peak, a double-resonant second order mode activated by in plane breathing of the hexagonal rings.^28^ The 2D/G Raman band intensity ratio was mapped over a mesoscopic area (Figure 1d) and takes values typically larger than one. The observed pattern reflects the variety of morphological configurations in the NPG sample, as discussed in ref (19). Both the low *I*_D_/*I*_G_ value and the fact that *I*_2D_/*I*_G_ > 1 are fingerprints of high quality graphene specimens. A typical C 1s spectrum (see Experimental and Computational Methods) acquired on the sample is shown in Figure 1e, showing the expected narrow peak at 284.3 eV binding energy (BE) with skewed line shape associated with the semimetallic graphene signal with a planar sp^2^ hybridization, without any defect-derived or contaminant-associated component.^19,20^ The micro-XPS mapping of the whole C 1s signal at the mesoscopic scale (Figure 1f) reveals the same tubular and continuous morphology observed in the SEM image (Figure 1b). This suspended 3D graphene continuous architecture can thus provide a novel route to obtain a 3D “graphane” structure, preventing interface/edge effects or defect induced adsorption.

**Figure 2:**
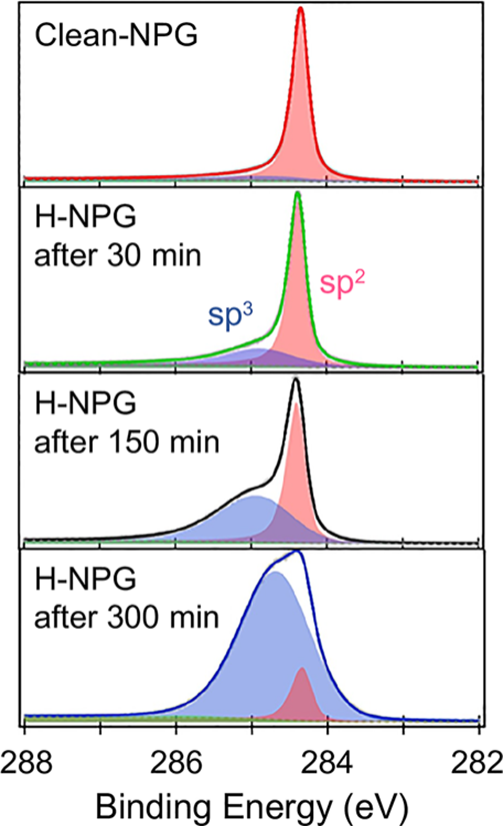
C 1s core level XPS spectra of UHV-clean NPG (top), and after atomic H exposure as a function of the exposure time, up to H saturation (300 min); experimental data (solid lines), sp^2^ (pinkish peaks) and sp^3^ (bluish peaks) fitting components.

Hydrogen uptake can be identified by the distortion of the pristine C–C sp^2^ bonds with formation of direct C–H sp^3^ bonds, as can be deduced by the line shape evolution of the C 1s core levels. Figure 2 displays the C 1s XPS core level spectra recorded for increasing hydrogen exposure up to saturation, along with the results of a fitting analysis carried out by using Voigt line shape curves (i.e., convolutions of a Lorentzian and a Gaussian distribution). The Lorentzian and Gaussian components are associated with the intrinsic core–hole lifetime and overall experimental resolution, respectively. After the first hydrogen exposure (30 min), a broad component at about 0.6 eV higher BE appears and increases in intensity as a function of the H dose. This component is related to the distortion of the sp^2^ bonding toward a sp^3^ hybridization of the C atoms. It is worth noting the absence of C 1s components due to unsaturated C bonds at lower binding energy,^8,29−33^ ensuring that a nondestructive and nondamaging hydrogenation process took place. Similarly, no oxidation component was found at higher BE,^34−36^ indicating that the high quality assessed for the clean NPG specimen was preserved. Longer H exposures until 300 min make the sp^3^ component dominant up to a saturated configuration, and beyond that exposure the line shape does not change. Our estimate for the sp^3^ bond distortion in the saturated configuration is Θ ∼ 90%, where Θ = *I*(sp^3^)/[*I*(sp^2^) + *I*(sp^3^)].

This extraordinary and unprecedented hydrogen uptake, which is in line with a chemisorption model for graphane with almost each C sp^3^ being bound, has never been achieved experimentally before in fully free-standing low-defect graphene. In fact, only partially hydrogenated graphene was observed until now—either on transferred monolayer flakes^16,37^ or on substrate-supported graphene^5,8^—with maximum uptake Θ ∼ 36%^14^ and with a high level of contamination and defects. The present successful procedure can be ascribed both to the morphology of the self-suspended, free-standing and bicontinuous NPG host and to the ultraclean and highly controlled, fully-UHV hydrogenation procedure.

**Figure 3:**
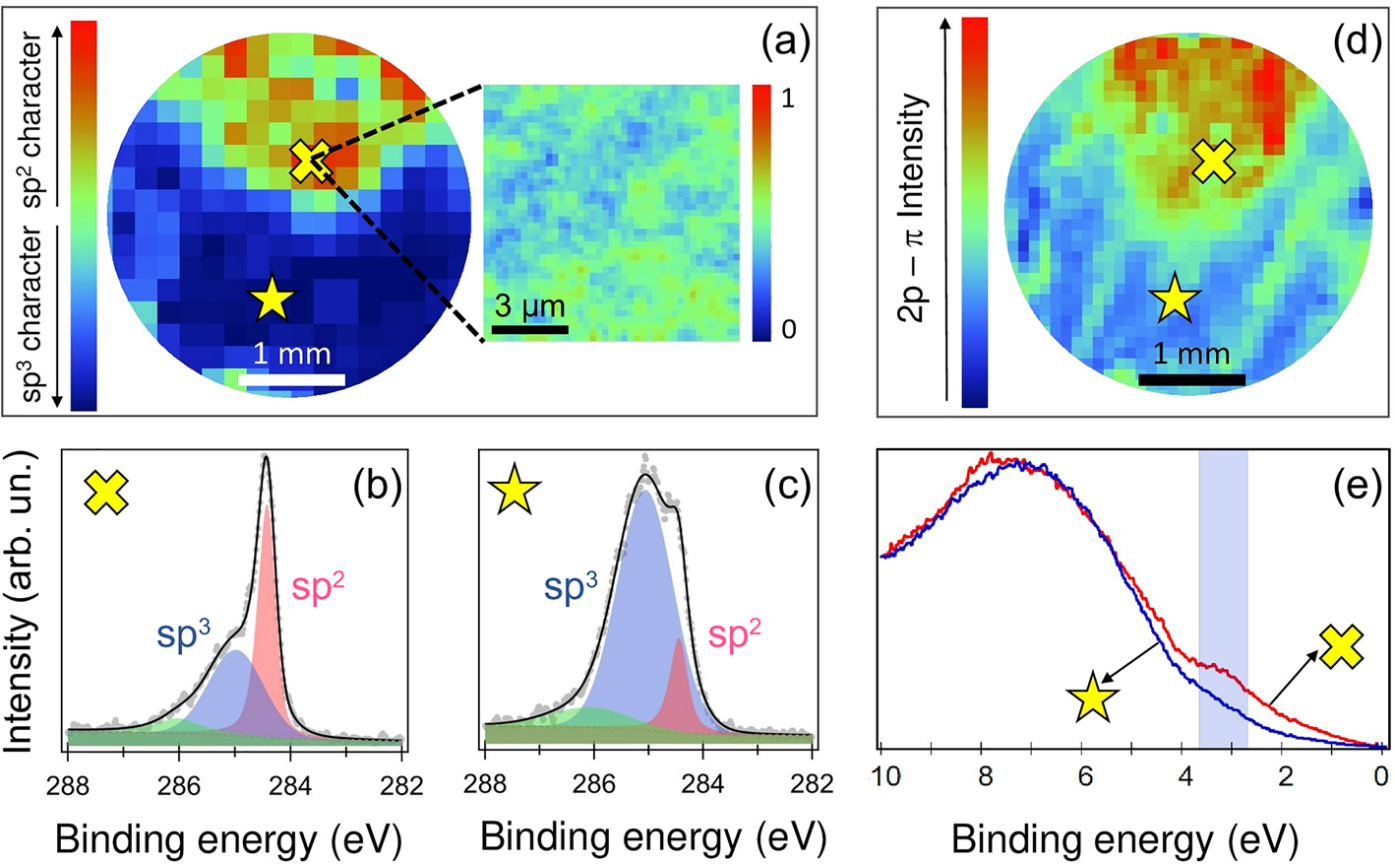
(a) C 1s intensity map, quantified as the ratio *I*_sp^2^_/(*I*_sp^2^_ + *I*_sp^3^_), after background subtraction; the blow-up represents the same ratio in a 10 × 10 μm^2^ area; the spectra taken in the sp^2^-rich and in sp^3^-rich regions (labeled by a cross and a star, respectively) are shown in panels (b) and (c), respectively. (d) Valence band (VB) intensity map corresponding to the 2p-π intensity. The ratio was calculated as in panel (a); the intensity was found by integrating in the energy range indicated by the shadowed vertical ribbon in panel (e), which displays the VB spectrum for sp^2^-rich (cross) and sp^3^-rich (star) regions.

A crucial point is to correlate the appearance of the distorted sp^3^ hybridization, due to the C–H bonding, to the opening of a semiconducting gap in graphane. Photoemission spectromicroscopy of both core levels and the valence band can combine local information on the hybridization state (core levels), with the evolution of the spectral density close to the Fermi level (valence band) at the same spatial scale. We measured a spatially resolved photoemission mapping of a partially hydrogenated NPG sample (Θ ≈ 50%), as to be able to identify regions with different degrees of hydrogenation, correlating valence band with core level spectral shapes. Figure 3a displays the spatially resolved intensity of the sp^2^ core-level component normalized to the whole sp^2^ + sp^3^ signal, as deduced by the fitting analysis (see Supporting Information) over the whole hydrogenated NPG specimen. This intensity varies in the map depending on the local hydrogenation state, as shown in the exemplifying spectra of Figure 3b,c. Areas where the sp^2^ hybridization is still dominant [cross in panel (a)] correspond to the C 1s spectrum displayed in panel (b), while zones with higher values of sp^3^ component [star in panel (a)] correspond to the C 1s spectrum in panel (c). The 10 × 10 μm^2^ blow up in Figure 3a shows that the normalized intensity ranges locally between 40 and 60%, thus suggesting a homogeneous hydrogenation at the local micrometer scale. A clear correlation with the core level spatial distribution can be observed by picking up the corresponding pixels in the valence band mapping, shown in Figure 3d: in the regions where the sp^2^ bond dominates, the spectral density of states presents a definite 2p-π peak at a binding energy of about 3 eV [see shaded area in panel (e)], which is the signature of the graphene band structure, while in the regions where the sp^3^ component emerges, the spectral density drastically changes and the 2p-π peak is almost quenched. It is worth noting that the density of states close to the Fermi level *E*_F_ is strongly reduced for all the pixels where the sp^3^ component dominates.

**Figure 4:**
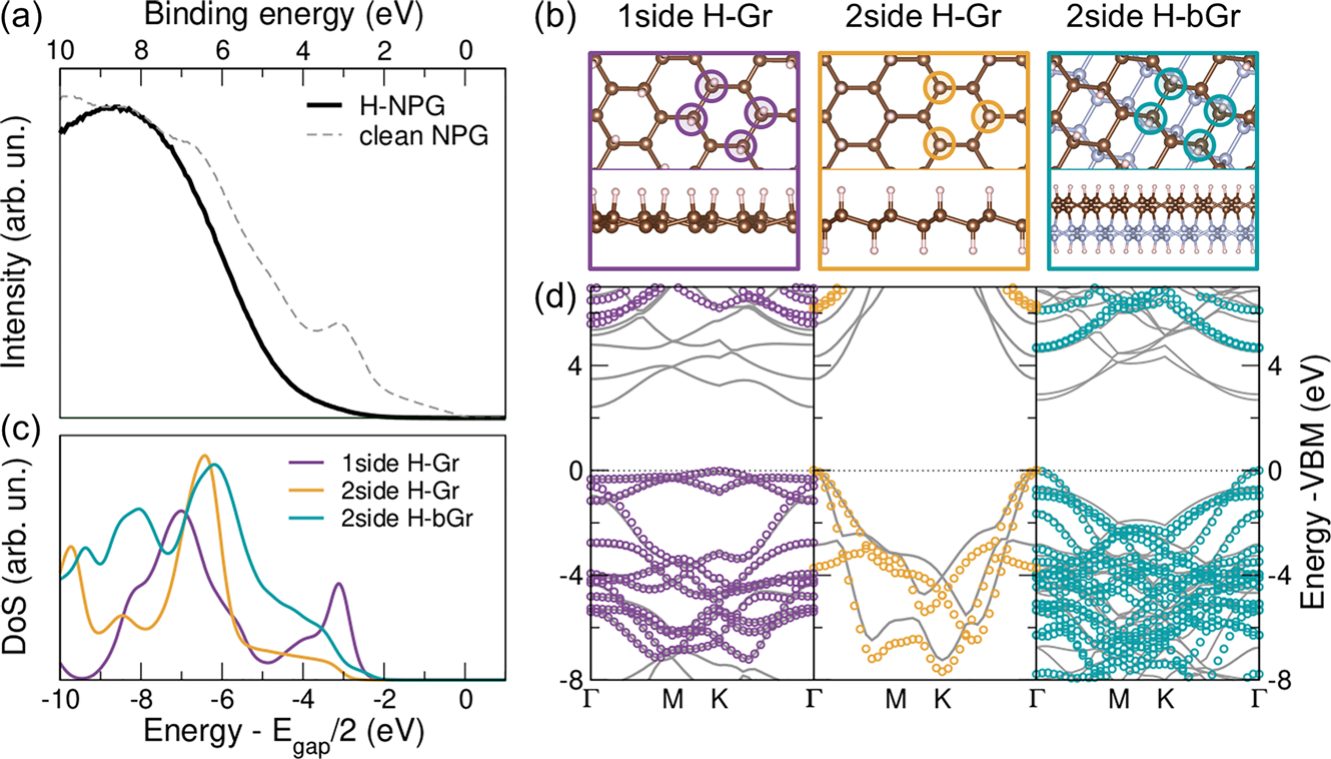
(a) Experimental VB spectra for clean (dashed) and totally hydrogenated NPG (solid line), taken with HeI_α_ (21.218 eV) photon energy. (b) Three model structures of 1-side and 2-side hydrogenated single- and bilayer graphene (top and side views). The colored circles in the top view highlight the H sites in the hexagon. (c) Simulated quasi-particle density of states (DoS) in the *GW* approximation for the models in (b); zero energy set at midgap; a homogeneous broadening of 140 meV is applied. (d) Simulated DFT (solid gray lines) and *GW* (open circles) band structures for the corresponding models in (b); zero energy set at the VBM.

At H saturation coverage (Θ ∼ 90*%*) the quenching of the density of states below *E*_F_ suggests a definite transition to a semiconducting state, as clearly observed in Figure 4a. In this novel configuration of the graphane band structure, the valence band maximum (VBM) can be extrapolated to be located at about 3.50 ± 0.25 eV below *E*_F_. The ascertainment of a definite correlation between the emergence of C–H sp^3^ bonding and the position of the VBM unambiguously ascribes the gap opening to the distortion of the bond to sp^3^, although the assignment of the hydrogen adsorption sites cannot be unambiguously identified from the photoemission experiment.

To corroborate and better understand the correlation between degree of H chemisorption and emergence of a wide-gap semiconducting phase, we simulated by means of density functional theory (DFT) calculations the spectral density for different hydrogenated graphene phases, including single-sided and double-sided hydrogenated single- and bilayer graphene, with different registry and hydrogenation configurations (details and configurations are reported in the Supporting Information). We hereafter report the results for three representative (the most stable ones) structures, that is (Figure 4b), single-sided hydrogenated graphene (1-side H-Gr or graphone^38^); 2-side H-Gr, i.e., graphane;^1,2^ and 2-side hydrogenated bilayer graphene (H-bGr). Quasi-particle corrections within the *G*_0_*W*_0_ approximation^39^ are included to overcome the DFT limitation in the description of the electronic properties and ease the comparison with experiments (see Experimental and Computational Methods). Figure 4 shows the quasi-particle density of states (DoS) (c) and band structures (d), after DFT geometric optimization. We find in all cases that the computed quasi-particle band gap for the free-standing hydrogenated sheets exceeds 3 eV (see Table S1 in Supporting Information). The single-sided hydrogenation leads to the appearance of an indirect gap of 5.6 eV. For the double-sided hydrogenation, we predict a direct gap of 4.7 eV for the H-bGr and 6.1 eV for H-Gr, the largest of the series, in line with previous calculations.^2^ For a more direct comparison with experiments, in Figure 4c we plot the DoS of the three systems, with the Fermi level set to midgap, a reasonable assumption given the high experimental quality (negligible contaminations/defects) of this hydrogenated sample.^40^ Irrespective of the exact position of the VBM onset, the single-sided H-Gr system noticeably presents a structured DoS at the VBM, originating from the 2p-π orbitals of the unsaturated side, that is totally absent in the experimentally achieved saturated phase of Figure 4a (solid thick line). On the contrary, the double-sided single- and bilayer hydrogenated graphene is characterized by a step-like DoS at the VBM, typical of 2D semiconductors,^41^ without any 2p-π contribution, in excellent agreement with the experimental data. Most of the spectral weight is indeed arising from the sp^3^ hybrid orbitals, lying in the energy region below −6 eV. This compares well in terms of energies and overall shape with the experimental spectrum (Figure 4a, solid thick line), taking into account the coexistence of single-layer and bilayer graphene^19,20^ in our NPG samples (see also Figure S1b). The experimental curve is, however, mostly featureless, probably due to the specific spectral amplitudes in this energy range,^42^ not accounted for in our calculations. Overall, we can conclude that the calculated band structure and quasi-particle DoS unequivocally allow us to establish the achievement of double-sided hydrogenated single- and bilayer graphene configurations.

## Conclusions

The smoothly rippled surface of nanoporous graphene, with very low density of defects, can foster a complete conversion in graphane, with almost all saturated C–H bonds, thanks to a carefully monitored in situ hydrogenation in ultrahigh vacuum conditions. Low-damage hydrogen deposition for long time exposures ensures an unprecedented atomic H uptake, with almost all available C sites saturated with H, as detected by the sp^3^ component in the C 1s core level, and a negligible presence of unsaturated bonds or defects.

Photoemission spectromicroscopy unveils a semiconducting band gap opening—correlated to the sp^3^ distorted C bonds—that the predicted spectral electronic density of states associates with the realization of double-sided, fully hydrogenated single- and bilayer structures, thus confirming this as a successful strategy to realize a thermodynamically stable prototype of graphane. Most interestingly, both single-layer and bilayer doubled-sided hydrogenated graphene unveil a direct band gap opening, which makes this prototype of semiconducting graphane a promising platform for optoelectronics applications.

## Experimental and Computational Methods

### Sample Preparation

Nanoporous graphene was synthesized by using a nanoporous Ni template via chemical vapor deposition (CVD). Ni_30_Mn_70_ ingots have been prepared by melting both pure metals in an Ar-protected arc melting furnace, then annealing at 900 °C to allow them to become microstructured and composition homogeneous, and rolling into thin films. The nanoporous Ni template was obtained from the ingot sheet by using chemical dealloying, before CVD grew graphene by using benzene as the carbon source. The as-grown NPG acquires the three-dimensional morphology of the substrate and is subsequently exfoliated by chemical dissolution of the Ni template. A detailed description of the process is described elsewhere.^24,43−46^ Hydrogenation has been done in situ in UHV by exposing NPG to atomic H produced by H_2_ cracking into a capillary source locally heated at 2100 °C.

### Photoemission Spectromicroscopy

The spectromicroscopy photoemission experiments were performed at the ANTARES beamline of the SOLEIL synchrotron radiation facility. The nano-X-ray photoelectron spectroscopy (XPS) microscope is equipped with two Fresnel zone plates for beam focusing, whereas higher diffraction orders were eliminated, thanks to an order selection aperture. The sample was placed on a precision positioning stage located at the common focus point of the hemispherical analyzer and the Fresnel zone plates. This setup was used for the collection of both point-mode spectra and imaging-mode spectra. In the imaging mode, the photoemitted electron intensity from the desired energy range is collected over the sample to form a 2D image resolved at the submicrometer range. Core-level and valence band spectra were taken with 350 and 100 eV photon energy, respectively. The spatial resolution was in the submicrometer range (near 600–700 nm). The analyzer pass energy was set to 100 eV (200 eV) for the spatially unresolved (resolved) mode. All measurements were made under ultrahigh vacuum (10^–10^ mbar), and prior to the acquisition the NPG samples were degassed at 600°C for several hours to remove contaminants from air exposure. The sample was kept cooled at the liquid nitrogen temperature to avoid radiation damage.^19^ Valence band data with 21.22 eV photon energy on the saturated H-NPG sample was taken at the Lotus laboratory, Rome, by using an analogous UHV setup.

### Raman Measurements

For Raman measurements, the excitation laser was provided by single frequency Nd:YVO_4_ lasers (DPSS series by Lasos) emitting at 532 nm. The Raman signal was spectrally dispersed by a 750 mm focal length ACTON SP750 monochromator equipped with a 300 groove/mm grating and detected by a back-illuminated N_2_-cooled Si CCD camera (model 100BRX by Princeton Instruments). The laser light was filtered out by a very sharp long-pass Razor edge filter (Semrock). The micro-Raman (μ-Raman) spectral resolution was 2.4 cm^–1^. A long working distance 100× objective with NA = 0.75 was employed to excite and collect the light, in a backscattering configuration. The laser spot size determined experimentally is characterized by a Gaussian shape with σ = 0.28 μm. Moderate laser powers (∼200 μW, corresponding to power densities ∼20 kW/cm^2^) were employed, after checking that the spectrum was analogous to that acquired with much lower power (a factor of ∼70).

### Theoretical Modeling

The ground-state properties of H-passivate graphene were investigated from first-principles by using a plane-wave pseudopotential implementation of the density functional theory (DFT), as available in the Quantum ESPRESSO package.^47,48^ The Perdew–Burke–Ernzerhof (PBE) generalized gradient approximation for the exchange-correlation functional was used,^49^ together with Optimized Norm-Conserving Vanderbilt (ONCV) pseudopotentials.^50^ The quasiparticle band structure for the DFT optimized geometries was computed within the *GW* approximation to the electron self-energy (*G*_0_*W*_0_ scheme;^39^ plasmon-pole model;^51^ slab truncation scheme for the Coulomb potential,^52^ random integration method for the screening potential), as implemented in the yambo code.^53,54^ Calculations were performed by employing an automated yambo-AiiDA based workflow.^55−57^

## Supporting Information Available

Details on the image analysis methods of the spectromicroscopy maps and details on the first-principles simulations (PDF)
