# Supplementary material for: Gap Opening in Double-Sided Highly Hydrogenated Free-Standing Graphene
Source: Nano Lett. 2022 Mar 16;22(7):2971–7. doi: 10.1021/acs.nanolett.2c00162 (PMC9011389; doi:10.1021/acs.nanolett.2c00162)
Supplement: Supplementary file 1 — nl2c00162_si_001.pdf [file nl2c00162_si_001.pdf]

SUPPORTING INFORMATION for Gap Opening in Double-Sided Highly Hydrogenated Free-Standing Graphene

Maria Grazia Betti,[*,†] Ernesto Placidi,[†] Chiara Izzo,[†] Elena Blundo,[†] Antonio Polimeni,[†] Marco Sbroscia,[†] José Avila,[‡] Pavel Dudin,[‡] Kailong Hu,[¶] Yoshikazu Ito,[§] Deborah Prezzi,[*,‖] Miki Bonacci,[⊥,‖] Elisa Molinari,[⊥,‖] and Carlo Mariani[†]

†*Physics Department – Sapienza University of Rome, Piazzale Aldo Moro 5, 00185 Rome (Italy)*

‡*Synchrotron SOLEIL, Université Paris-Saclay, Saint Aubin, BP 48, 91192 Gif sur Yvette (France)*

¶*School of Materials Science and Engineering, and Institute of Materials Genome & Big Data, Harbin Institute of Technology, Shenzhen 518055 (P.R. China)*

§*Institute of Applied Physics, Graduate School of Pure and Applied Sciences, University of Tsukuba, Tsukuba 305-8573 (Japan)*

‖*S3, Istituto Nanoscienze-CNR, Via Campi 213/A, 41125 Modena (Italy)*

⊥*Dipartimento di Scienze Fisiche, Informatiche e Matematiche (FIM), Università degli Studi di Modena e Reggio Emilia, 41125, Modena (Italy)*

E-mail: mariagrazia.betti@uniroma1.it; deborah.prezzi@nano.cnr.it

Phone: +39 06 49914389; +39 059 2055314

1 Image analysis

The spatially resolved photoemission spectromicroscopy measurements taken at the Antares beamline of the Soleil synchrotron radiation facility (France), are constituted by a matrix of pixels where each pixel contains the full spectrum (spectral density versus binding energy). The plots displayed in Figure 3 (main text) were created according to the following procedure:

- XPS core-level and VB spectra were extracted from each pixel by means of a macro routine, and all the spectra were recorded on separate files.
- All core-level spectra were fitted with KolXPD software taken fixed the energy position of the components (sp^2, sp^3, CO_x) and their Lorentzian width, leaving free only the Gaussian width. All the areas of the different components (including the background area) were recorded and registered as matrix text files. For the VB data only the spectral density intensity in the BE region across the 2p-π peak was considered.
- The images reported in Figure 3 were created from the spectral matrix files with Gwyddion software, after normalization (dividing the sp^2 intensity by the sum of (sp^2+sp^3); an analogous procedure subtracting the background from the 2p-π peak energy region was used for the VB image.

2 First principles simulations

The ground-state properties of H-passivate graphene were investigated from first principles by using a plane-wave pseudopotential implementation of the density functional theory (DFT), as available in the Quantum ESPRESSO package.[1,2] The Perdew-Burke-Ernzerhof (PBE) generalized gradient approximation for the exchange-correlation functional was used,[3] together with Optimized Norm-Conserving Vanderbilt (ONCV) pseudopotentials.[4]

We considered different configuration, displayed in Figure S1a, investigating both single- (1side) and double-side (2side) hydrogenation of the single- (H-Gr) and bi-layer (H-bGr)

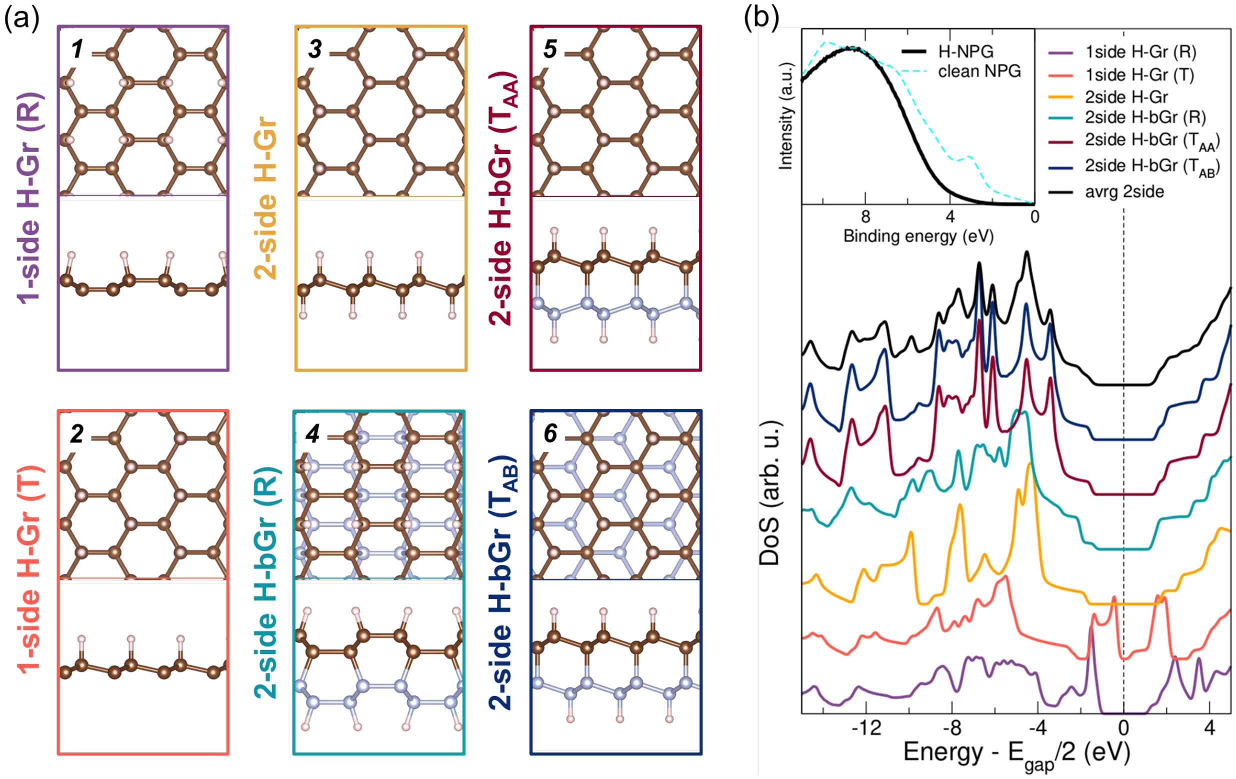

Figure S1: (a) Ball-and-stick models (top and side views) for different hydrogenated graphene structures (1-6). C (H) is represented by brown (white) spheres. For the bilayer structures (4-6), the bottom C layer is represented in light blue, in order to make the figures clearer. (b) DFT-PBE density of states (DoS) for the configurations reported in (a). An additional curve, representing the average of all the 2-side hydrogenated configurations (avrg 2side, solid black line), is also reported in the main panel, to ease the comparison with the experimental VB spectra for clean (inset, dashed cyan) and totally hydrogenated NPG (inset, solid thick black line), taken with HeI$_\alpha$ (21.218 eV) photon energy.

graphene basal plane. In fact, in addition to the ideal *graphane* configuration (Figure S1a-3), where each C atom of single-layer graphene is bound to H forming an alternately up and down sp^3 distorted structure (also called *chair* or meta), we also considered the case of single-layer graphene saturated on one side only, i.e. the so-called *graphone.*[5] For the latter, we investigated both the *chair-like* (Figure S1a-2), also denoted as triangular, T, or meta) and *boat-like* (Figure S1a-1, also denoted as rectangular, R, or para/ortho) structures, the latter being the most stable one for single-side hydrogenated free-standing graphene.

Moreover, given the presence of few-layer graphene with misoriented (turbostratic) stacking in some regions, as recognised by an atomic-scale analysis of the NPG microscopic structure,[6] we also considered the two-side hydrogenation of graphene bilayers with different registry, in order to explore possible local structures of a turbostratic arrangement, as displayed in Figure S1a(4-6). The 2-side hydrogenation of the bilayer graphene is found to forster the formation of C puckering and interlayer bonding, as also found in the studies on the stability and realization of diamanes, i.e. 2D diamond-like thin films, as summarized in a recent review.[7]

For each of the above described systems, the atomic positions within the cell were fully relaxed until forces were less than 5×10^{-4} a.u. A vacuum region larger than 15 Å in the non-periodic direction was introduced to prevent interaction between periodic images. The kinetic energy cutoff for the wave functions was set to 80 Ry; the Brillouin zone was sampled by using a $14 \times 14 \times 1$ ($8 \times 8 \times 1$) **k**-points grid for the primitive (2×2) cell, respectively, according to Monkhorst-Pack algorithm. The optimized in-plane lattice parameters, $a = b$, are reported in S1, together with the C-C bond lengths, l_{CC}, as well as the energy gap $E_{g,PBE}$. Figure S1b displays the DFT-PBE density of states (DoS) for the configurations described above and reported in panel (a). An additional curve, representing the average of all the 2-side hydrogenated configurations (solid black line), is also reported in panel (b), to mimic the results for turbostratic few-layer samples and ease the comparison with the experimental VB spectra (inset, dashed cyan and solid thick black lines). While the comparison clearly

highlights the well-known limitations of the DFT-PBE theory for a quantitative description of the electronic properties,[8] the overall shape is in agreement with experimental findings. The theory curve is however much more structured if compared to the featureless curve of H-NPG presented in the inset. This is because we only apply a homogeneous broadening of 140 meV to build our DoS, while experimental spectra contain information on the the real spectral amplitude, which is an energy and k-dependent value.[9]

Table S1: Calculated structural and electronic properties of the different H-Gr configurations displayed in Table S1. Except for the 2side H-Gr, all systems are computed in a 2×2 supercell.

System	a (Å)	l_{CC} (Å)	$E_{g,PBE}$ (eV)	E_{g,G_0W_0} (eV)
1side H-Gr (T)	5.063	1.50	0.65 (I)	3.18
1side H-Gr (R)	5.019	1.36; 1.50; 1.57	2.46 (I)	5.60
2side H-Gr	2.537	1.54	3.49	6.15
2side H-bGr (R)	4.960	1.61; 1.56; 1.53; 1.57	2.69	4.70
2side H-bGr (T_{AA})	5.043	1.54; 1.58	2.90	-
2side H-bGr (T_{AB})	5.057	1.54; 1.56	3.10	5.10

To overcome some of the above-mentioned limitations, the quasiparticle band structure and density of states (DoS) for selected DFT optimized geometries were computed within the GW approximation to the electron self-energy (G_0W_0 scheme, plasmon-pole model[10]), as implemented in the YAMBO code.[11,12] A truncation scheme[13] for the Coulomb potential was adopted to avoid spurious interactions between replicas. The Brillouin zone was sampled by $16 \times 16 \times 1$ **k**-points for the primitive cell. We used a random integration scheme for the calculation of the screened Coulomb interaction, ensuring convergence of the quasiparticle gap with such low density **k**-points meshes. The sum-over-states in the calculation of polarization function and Green function have been truncated both at 800 bands. The kinetic energy cutoff to represent the response functions corresponds to 30 Ry. The above parameters were chosen by considering a convergence threshold below 15 meV on the fundamental gaps, by employing an automated yambo-AiiDA based workflow.[14–16] The energy gap values

E_{g,G_0W_0} computed according to the G_0W_0 approximation are reported in Table S1, while the DoS and full bandstructures of selected cases are reported in Figure 4 of the main text.

References

(1) Giannozzi, P. et al. QUANTUM ESPRESSO: a modular and open-source software project for quantum simulations of materials. *J. Phys.-Condens. Mat.* **2009**, *21*, 395502.

(2) Giannozzi, P. et al. Advanced capabilities for materials modelling with Quantum ESPRESSO. *J. Phys.-Condens. Mat.* **2017**, *29*, 465901.

(3) Perdew, J. P.; Burke, K.; Ernzerhof, M. Generalized gradient approximation made simple. *Phys. Rev. Lett.* **1996**, *77*, 3865.

(4) Hamann, D. R. Optimized norm-conserving Vanderbilt pseudopotentials. *Phys. Rev. B* **2013**, *88*, 085117.

(5) Zhou, J.; Wang, Q.; Sun, Q.; Chen, X. S.; Kawazoe, Y.; Jena, P. Ferromagnetism in Semihydrogenated Graphene Sheet. *Nano Letters* **2009**, *9*, 3867–3870.

(6) Di Bernardo, I.; Avvisati, G.; Mariani, C.; Motta, N.; Chen, C.; Avila, J.; Asensio, M. C.; Lupi, S.; Ito, Y.; Chen, M.; Fujita, T.; Betti, M. G. Two-Dimensional Hallmark of Highly Interconnected Three-Dimensional Nanoporous Graphene. *ACS Omega* **2017**, *2*, 3691–3697.

(7) Chernozatonskii, L. A.; Demin, V. A.; Kvashnin, D. G. Fully Hydrogenated and Fluorinated Bigraphenes-Diamanes: Theoretical and Experimental Studies. *C* **2021**, *7*.

(8) Onida, G.; Reining, L.; Rubio, A. Electronic excitations: density-functional versus many-body Green's-function approaches. *Rev. Mod. Phys.* **2002**, *74*, 601–659.

(9) Marzari, N.; Ferretti, A.; Wolverton, C. Electronic-structure methods for materials design. *Nat. Mater.* **2021**, *2*, 736 – 749.

(10) Godby, R. W.; Needs, R. J. METAL-INSULATOR-TRANSITION IN KOHN-SHAM THEORY AND QUASIPARTICLE THEORY. *Phys. Rev. Lett.* **1989**, *62*, 1169–1172.

(11) Marini, A.; Hogan, C.; Grüning, M.; Varsano, D. yambo: An ab initio tool for excited state calculations. *Comp. Phys. Commun.* **2009**, *180*, 1392–1403.

(12) Sangalli, D. et al. Many-body perturbation theory calculations using the yambo code. *J. Phys.: Condens. Matter* **2019**, *31*, 325902.

(13) Rozzi, C. A.; Varsano, D.; Marini, A.; Gross, E. K. U.; Rubio, A. Exact Coulomb cutoff technique for supercell calculations. *Phys. Rev. B* **2006**, *73*, 205119.

(14) Huber, S. P. et al. AiiDA 1.0, a scalable computational infrastructure for automated reproducible workflows and data provenance. *Sci. Data* **2020**, *7*, 300.

(15) Uhrin, M.; Huber, S. P.; Yu, J.; Marzari, N.; Pizzi, G. Workflows in AiiDA: Engineering a high-throughput, event-based engine for robust and modular computational workflows. *Comp. Mat. Sci.* **2021**, *187*, 110086.

(16) The yambo-AiiDA code is available at https://github.com/yambo-code/yambo-aiidahttps://github.com/yambo-code/yambo-aiida.
